# Analysis of Serum Fatty Acids Profile in Kidney Transplant Recipients

**DOI:** 10.3390/nu13030805

**Published:** 2021-02-28

**Authors:** Adriana Mika, Lukasz P Halinski, Tomasz Sledzinski, Sylwia Malgorzewicz, Paulina Woloszyk, Jolanta Dardzinska, Alicja Debska-Slizien, Michal Chmielewski

**Affiliations:** 1 Department of Pharmaceutical Biochemistry, Medical University of Gdansk, Debinki 1, 80-211 Gdansk, Poland; adriana.mika@gumed.edu.pl; 2 Department of Environmental Analysis, Faculty of Chemistry, University of Gdansk, Wita Stwosza 63, 80-308 Gdansk, Poland; lukasz.halinski@ug.edu.pl; 3 Department of Nephrology, Transplantology and Internal Medicine, Medical University of Gdansk, Debinki 7, 80-211 Gdansk, Poland; sylwia.malgorzewicz@gumed.edu.pl (S.M.); adeb@gumed.edu.pl (A.D.-S.); chmiel@gumed.edu.pl (M.C.); 4 Department of Clinical Nutrition, Medical University of Gdansk, Debinki 7, 80-211 Gdansk, Poland; jolanta.dardzinska@gumed.edu.pl; 5 Department of Pediatric and f Internal Nursing, Medical University of Gdansk, Debinki 7, 80-211 Gdansk, Poland; paulina.woloszyk@gumed.edu.pl

**Keywords:** cardiovascular disease, diabetes mellitus, fatty acids, GC-MS, kidney transplantation, lipids, PCA

## Abstract

Patients with end-stage kidney disease, treated with renal transplantation, are at increased risk of cardio-vascular disease (CVD) and cardio-vascular mortality. They are also characterized by an atherogenic dyslipidemia. Alterations of the fatty acids (FA) profile contribute to increased cardio-vascular risk in the general population. In the current study we test the hypothesis that kidney transplantation is associated with ab-normalities in FA profile. FA profile was analysed by gas chromatography–mass spectrometry in 198 renal transplant recipients, and 48 control subjects. The most profound differences between renal transplant patients and controls were related to the content of branched chain FA, monounsaturated FA, and n-6 polyunsaturated FA, respectively. The FA profile significantly separated the patients from the controls in the principal component analysis (PCA). The abnormalities of FA profile showed a tendency for normalization in long-term kidney recipients, as compared to patients with recent transplants. The n-3 PUFA content demonstrated a strong inverse association with the presence of inflammation. Most profound alterations of the FA profile were observed in patients with impaired graft function (glomerular filtration rate < 45 mL/min). The study demonstrated significant disorders of the FA profile in kidney transplant recipients, that might contribute to cardio-vascular risk in this vulnerable patient population.

## 1. Introduction

Chronic kidney disease (CKD) is a fairly common condition with a prevalence of 5–15% of the general population [1,2]. This, already high prevalence is still on the rise, in association with increasing lifespan and diseases that contribute to CKD, as diabetes mellitus, hypertension and atherosclerosis. Patients with CKD are at increased risk of cardiovascular disease (CVD) and cardiovascular complications [3]. This risk is known to increase with CKD progression. In the general population, CVD is heavily dependent on lipid disturbances. This, to some extent, could also be the case in patients with renal failure, as dyslipidemia is a constant feature of CKD [4]. The most prevalent lipid disorders in CKD include increased concentration of triglycerides, and low level of high-density lipoproteins (HDL) cholesterol, two acknowledged risk factors for CVD in the general population. However, not only these lipid fractions are disturbed in the course of CKD. Our previous studies demonstrate that the profile of fatty acids (FA) is significantly affected by the disease [5,6,7]. Decreased content of polyunsaturated fatty acids (PUFA), together with a high level of endogenously generated monounsaturated fatty acids (MUFA) are thought to contribute to the increased CVD risk both, in the general population and in CKD subjects [8,9]. Kidney transplantation is the best treatment option for patients with the most advanced stage of CKD, end-stage renal disease (ESRD). The risk of cardiovascular complications decreases, as compared to patients treated with chronic dialysis. However, even in this patient group, cardiovascular events remain the major cause for mortality [10]. In our earlier studies, we have shown that despite normalization of kidney function parameters in renal transplant recipients, the FA profile is still altered [6,11]. The current evaluation aims at investigating in-depth the FA disorders in this patient population. The outcome under analysis was the content of serum fatty acids in renal transplant recipients in comparison to controls with no kidney insufficiency.

## 2. Materials and Methods

### 2.1. Patients

The study included 198 CKD patients, aged 18–70 years, with a functioning kidney graft, as well as an age- and sex-adjusted control group of 48 subjects without CKD. All patients after transplantation (Tx) were in a clinically stable condition and were treated with triple immunosuppressive protocols including cyclosporine or tacrolimus, mycophenolate mofetil and steroids. Plasma samples were collected after an overnight fast, and stored at −80 °C until analyzed. Patients using FA supplements were excluded from the study. The patients generally agreed to participate by donating blood for analysis, with a recruitment success percentage exceeding 90%. The study was performed in agreement with the principles of the Declaration of Helsinki of the World Medical Association. The study protocol received approval from the Local Bioethics Committee at the Medical University of Gdansk (protocol no. NKEBN/614/2013–2014 issued on 28 May 2014) and informed consents were obtained from patients and healthy volunteers. Estimated glomerular filtration rate (eGFR) was calculated with the CKD-EPI formula [12]. Presence of CVD, diabetes mellitus and hypertension was based on the medical records. The following laboratory parameters were measured: haemoglobin, creatinine, blood urea nitrogen (BUN), C-reactive protein (CRP), blood lipids, sodium, potassium by routine laboratory methods in clinical laboratory of our hospital. To assess the changes in FA profile with time after renal transplantation, the Tx group was subdivided into patients directly (up to one month) after the procedure (Tx 1), patients between 1 month and the first year post-transplant (Tx 12), and subjects in their consecutive years post-transplant (Tx > 12). Nutritional status was estimated by 7-SGA (7-point subjective global assessment), in which a score 7 and 6 indicated good nutritional status, 5, 4 and 3 mild malnourishment, while 2 and 1 severe malnourishment. Body composition was assessed by multi-frequency bioimpedance method (BCM Body Composition Monitor, Fresenius SA, Germany). It included an assessment of the lean body mass, fat mass, body water, fat tissue index (FTI), and lean tissue index (LTI). The biochemical and anthropometric characteristics of the study subjects are presented in Table 1.

### 2.2. Lipid Analysis

Extraction of total lipids from serum samples was performed with chloroform-methanol mixture (2:1, *v*/*v*) according to the method of Folch et al. [13]. Extracts were dried under nitrogen stream, reconstituted in 1 mL of 0.5 M KOH in methanol and hydrolysed at 90 °C. After 3 h the mixture was acidified with 0.5 mL of 6 M HCl, 1 mL of H_2_O was added and FAs were extracted with 3 volumes of 1 mL n-hexane. FAs were derivatized into fatty acids methyl-esters (FAMEs) via incubation with boron trifluoride-methanol solution at 55 °C for 90 min. Subsequently, 1 mL of H_2_O was added to the mixture and FAMEs were extracted with 3 volumes of 1 mL n-hexane. Solvent was then evaporated under nitrogen stream and samples were stored at −20 °C until analysis. Directly before analysis, samples were reconstituted in dichloromethane and FAMEs were analysed by GC-MS QP-2010 SE (Shimadzu, Kyoto, Japan). The separation was carried out on a Zebron ZB-5MSi capillary column (30 m length × 0.25 mm i.d. × 0.25 µm film thickness). Overall run time of the analysis was 60 min, and the column temperature was set between 60–300 °C (4 °C/min). The carrier gas was helium, with column head pressure of 100 kPa. The electron impact source for mass detection was operating at 70 eV. Mass spectra acquisition was carried out in full scan mode (m/z 45–700). 19-methylarachidic acid was used as an internal standard. FAs were identified using reference standards (37 FAME Mix, Sigma-Aldrich, Saint Louis, MI, US) and reference library NIST 2011.

### 2.3. Statistical/Chemometric Analysis

Data analysis was performed in Sigma Plot (Systat Software Inc., San Jose, CA, USA). Comparisons between two groups were done with two-tailed *t*-test (for parametric data) and Mann-Whitney Rank Sum Test (for non-parametric data). For 3 or more groups one way analysis of variance (ANOVA) was performed, followed by all pairwise multiple comparison procedures (Tukey test) for parametric data. Non-parametric data was subjected to Kruskal-Wallis ANOVA on ranks followed by Tukey test. All values are presented as mean ± SEM. The chemometric data analysis was carried out using the computing environment R [14]. Principal component analysis (PCA) was performed using the FactoMineR package [15] with the factoextra package for data visualization. Two-way hierarchical cluster analysis (HCA) was carried out using cim function of the mixOmics package [16]. Squared Euclidean distance was used as the measure of similarity, and Ward method was applied for the agglomeration. All data matrices were auto-scaled before the analysis.

## 3. Results

As expected, transplant recipients were characterized by hypertriglyceridemia and low HDL cholesterol, as compared to controls (Table 1). Comparisons between kidney transplant patients and healthy controls (HC) regarding profile of FA in serum of subjects were conducted using Student’s t test and principal component analysis (PCA). Numerous significant differences were observed between kidney transplant patients and healthy controls (HC) regarding profile of FA in serum (Table 2). In Tx patients, there were lower levels of long chain FA, straight chain fatty acids containing twelve or more carbon atoms (LCFA) (with the exception of palmitic acid (16:0) that was higher in Tx patients) and very long chain FA, fatty acids containing twenty or more carbon atoms (VLCFA), some representatives of odd chain fatty acid (OCFA), almost all iso and anteiso branched chain fatty acids (BCFA), cyclopropaneoctanoic acid 2-hexyl (CPOA2H), and almost all polyunsaturated fatty acids PUFA without docosahexaenoic acid (22:6n3; DHA), docosapentaenoic acid (22:5n3; DPAn-3) and adrenic acid (22:4n6; AdA). Only two major monounsaturated fatty acids (MUFA), including palmitoleic acid (16:1) and oleic acid (18:1) were observed in elevated levels in Tx patients, in comparison to the HC group. The remaining very long chain monounsaturated FA (VLC-MUFA) were lower in Tx patients compared with HC. Also, OCFA including 15:0, dominated in Tx, and another dietary FA, 17:0, presented a trend to be in higher levels in the Tx group (Table 2).

In order to exclude the short-time effect of surgery (transplantation) and accompanying inflammation, in the next analysis between these two groups, we excluded patients up to 3 months after kidney transplantation (Table S1). The smaller differences between HC and Tx as well as less significant differences were observed in case of LCFA and VLCFA. However, the other FA groups did not show large changes in composition in comparison to earlier analysis, which included all Tx patients (Table S1).

**Figure 1:**
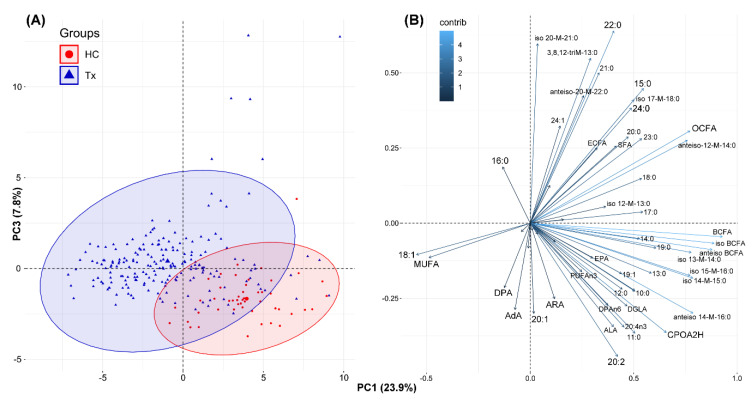
The results of PCA based on the serum fatty acid profiles: score plot of cases (**A**) and variables (**B**) for healthy control (HC) and Tx (transplant) patients. For statistical significance of differences between groups please consult Table 2. AdA-adrenic acid; ALA-α-linolenic acid; ARA-arachidonic acid; BCFA-branched chain fatty acids; CPOA2H-cyclopropaneoctanoic acid 2-hexyl; DGLA-dihomo-γ-linolenic acid; DHA-docosahexaenoic acid; DPA n3-docosapentaenoic acid n3; DPAn6-docosapentaenoic acid n6; ECFA-even chain fatty acids; EPA-eicosapentaenoic acid; ETA-eicosatetraenoic acid; LA-linoleic acid; MUFA-monounsaturated fatty acids, OCFA-odd chain fatty acids; PC1-principal component 1; PC3-principal component 3; PUFA-polyunsaturated fatty acids; SFA-saturated fatty acids.

The next analysis, PCA, showed, that the profile of FA in serum separated Tx patients and healthy subjects (Figure 1). Similar separation was also observed when patients up to 3 months after kidney transplantation were excluded from the dataset (Figure S1). Separation was mostly based on elevated 18:1 and decreased several SFAs in the serum of Tx patients, and much higher levels of branched FA, CPOA2H and selected PUFA in the serum of HC (see right lower part of the Figure 1B). This was consistent with the data shown in Table 2. The overall variation in FA composition was much higher in Tx patients and was described mostly by PC1 in PCA (see the size of blue ellipse in Figure 1A).

Also, in the analysis of serum FA profile in consecutive kidney transplant groups by ANOVA with the Tukey post hoc test, a multitude of significant differences were observed (Table 3). In general, most profound disturbances in the FA profile were noted early after transplantation, i.e., in the Tx1M group. The results in Tx12M and Tx>12M were comparable, demonstrating an early stabilization, and showed a trend towards normalization. However, they still differed from the values observed in HC. Comparison of the serum FA profile of Tx1M patients with the other Tx groups showed an increase in the VLCFA with odd and even aliphatic chains in patients over 1 month after transplantation. The total BCFA content in Tx>12M patients were significantly reduced, as compared to HC. The contents of the four major FA, palmitic acid (16:0), palmitoleic acid (16:1), oleic (18:1) and oleic acid (18:0) decreased with time after transplantation. As for PUFA, we noted significant increase of their amount with time after transplantation, with PUFA contents in the Tx>12M group comparable to controls (Table 3).

**Figure 2:**
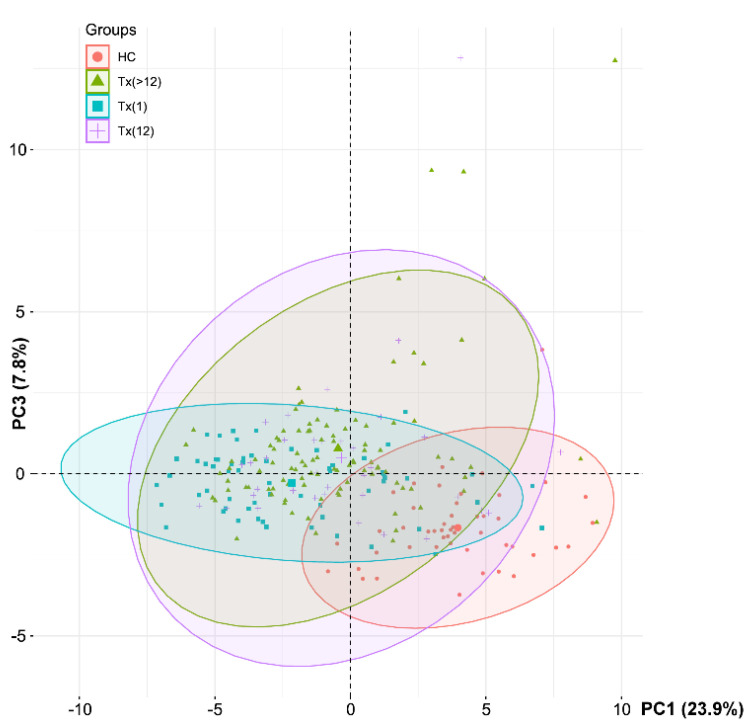
The results of principal component analysis (PCA) of individuals based on the whole serum fatty acid profile. See Figure 1 for the plot of variables. HC – healthy controls; Tx(1)-patients up to one month after the procedure; Tx (12)-patients between 1 month and the first year post-transplant; Tx (>12)-patients in their consecutive years post-transplant.

PCA showed slight separation of HC group from Tx groups, but the overall FA profile was not different among the individual Tx groups (Figure 2). Surprisingly, the FA profile was more consistent within the group of patients just after the transplantation.

**Figure 3:**
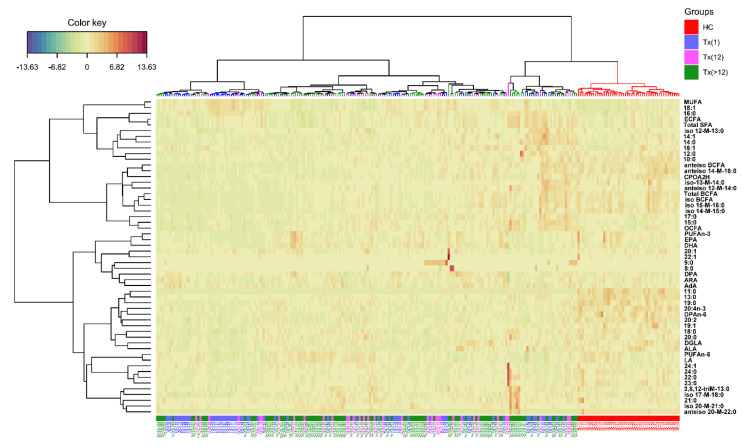
Heatmap generated basing on the serum profile of 55 fatty acids in healthy control (HC, red) and Tx patients. Rows: fatty acids; columns: individuals. Color key indicates fatty acid expression value: dark blue: lowest; dark red: highest. HC – healthy controls; Tx(1)-patients up to one month after the procedure; Tx (12)-patients between 1 month and the first year post-transplant; Tx (>12)-patients in their consecutive years post-transplant. AdA-adrenic acid; ALA-α-linolenic acid; ARA-arachidonic acid; BCFA-branched chain fatty acids; CPOA2H-cyclopropaneoctanoic acid 2-hexyl; DGLA-dihomo-γ-linolenic acid; DHA-docosahexaenoic acid; DPAn6-docosapentaenoic acid n6; DPA-docosapentaenoic acid n3; ECFA-even chain fatty acids; EPA-eicosapentaenoic acid; ETA-eicosatetraenoic acid; LA-linoleic acid; MUFA-monounsaturated fatty acids, OCFA-odd chain fatty acids; PUFA-polyunsaturated fatty acids; SFA-saturated fatty acids.

Heatmap based on the FA profiles, as a result of a two-way hierarchical cluster analysis (HCA) of biomarkers, was used to assess relatedness of different FA and visually presentation of the diversity of samples. The heatmap showed that Tx patients and HC were almost completely separated from each other. It could be noted that the metabolic state of Tx patients resulted in the decreased levels of n-3 and n-6 PUFA, iso and anteiso BCFA and VLCFA, as well as elevated levels of MUFA. Further, The result of HCA showed that these FA could distinguish the Tx patients after 1 month and other Tx patients (Figure 3). Surprisingly, there was a small number of patients from Tx12M and Tx>12M groups, that was significantly different from other patients in terms of much higher levels of long-chain SFA, MUFA and branched FA (Figure 3).

**Figure 4:**
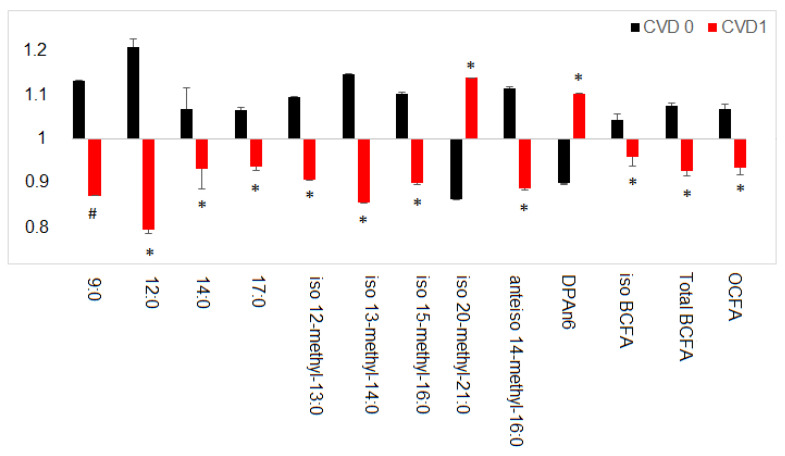
Normalized FA differences in Tx patients with (CVD 1) or without (CVD 0) cardiovascular disease. Data are presented as mean ± SEM. * *p* ˂ 0.05; # *p* ˂ 0.001 indicates statistically significant difference compared to CVD 0. BCFA-branched chain fatty acids; DPA n6-docosapentaenoic acid n6; OCFA-odd chain fatty acids.

When analyzing the association between the FA profile and concomitant diseases of transplanted patients, significant differences were observed between subjects with and without CVD, as demonstrated in Figure 4.

The amounts of medium and long chain FA, generally regarded as proinflammatory, including 9:0, 12:0 and 14:0, were increased, while dietary FA acknowledged as salutary, including 17:0 and many representatives of BCFA, were significantly reduced. Moreover, we found an increased level of the proinflammatory docosapentaenoic acid (22:5n6; DPAn6) (Figure 4).

**Figure 5:**
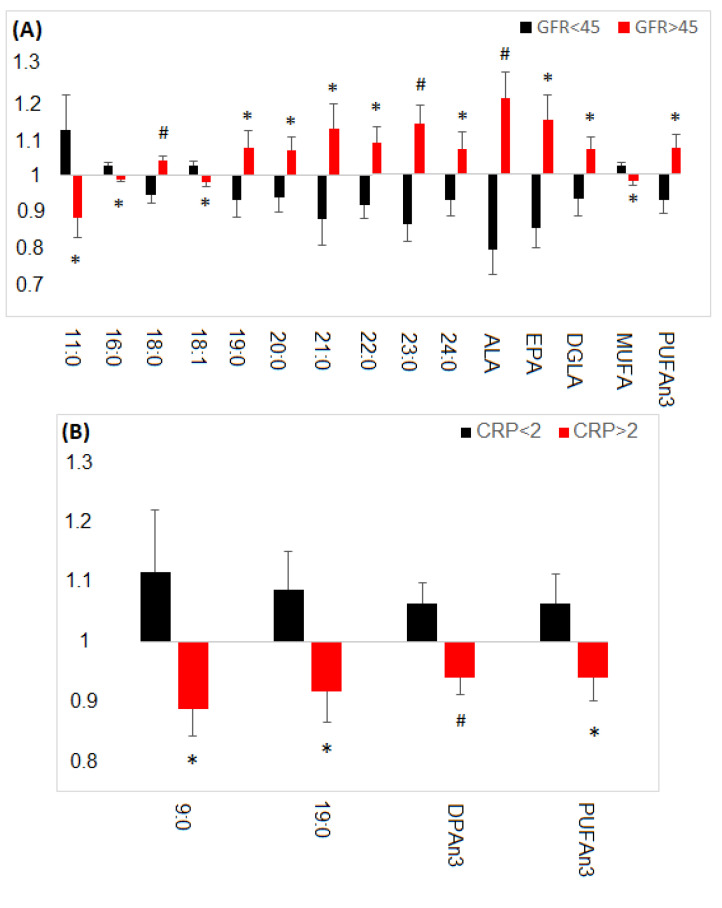

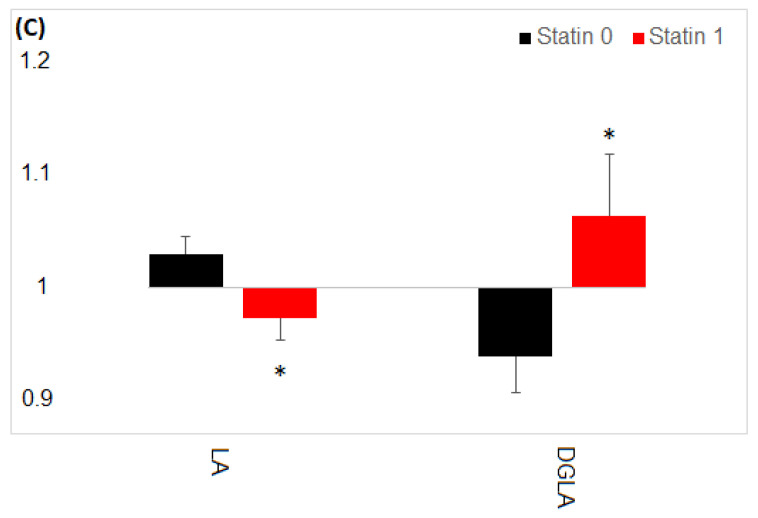
Normalized FA changes in different subgroups of Tx patients (**A**) With glomerular filtration rate below and above 45; (**B**) With c-reactive protein below and above; (**C**) Using (Statin 1) or no using statins (Statin 0). Data are presented as mean ± SEM. * *p* ˂ 0.05; # *p* ˂ 0.001 indicates statistically significant difference compared to controls. ALA-α-linolenic acid; DGLA-dihomo-γ-linolenic acid; DPA n3-docosapentaenoic acid n3; EPA-eicosapentaenoic acid; LA-linoleic acid; MUFA-monounsaturated fatty acids; PUFA-polyunsaturated fatty acids.

Grafted kidney function, as determined by the glomerular filtration rate (GFR) also strongly differentiated the FA profile of the studied patients (Figure 5A). In patients with impaired renal function (GFR < 45 mL/min/1.73 m^2^) we observed significantly lower content of PUFA, including eicosapentaenoic acid (20:5n3; EPA) and α-linoleic acid (18:3n3; ALA), and dihomo-γ-linolenic acid (20:3n6; DGLA). In turn, 18:1 and the total content of MUFA was elevated in Tx patients with worse kidney function (Figure 5A). Finally, majority of the VLCFA were reduced in Tx patients with GFR<45 mL/min/1.73 m^2^, and shorter saturated FA (11:0 and 16:0) were increased in this subgroup (Figure 5A).

Inflammation, determined by CRP above 2 mg/L, was associated with reduced levels of n3DPA and total n3PUFA, as well as with reduced 9:0 and 19:0 levels (Figure 5B). An interesting relationship was observed in the case of Tx patients using statins (Figure 5C). The level of linoleic acid (18:2n6, LA), which is precursor of DGLA, was reduced in Tx patients, who were taking statins, and DGLA, was elevated.

Time after transplantation, CVD, inflammation, and impaired graft function were associated with the most profound changes in the FA profile in patients after kidney transplantation (Figure 4; Figure 5). Other parameters including, body mass index (BMI), fat tissue index (FTI), lean tissue index (LTI), nutritional status (7-SGA), dialysis before transplantation, type of immunosuppressive therapy, supplementation with vitamin D3, angiotensin-converting enzyme (ACE) inhibitors, were also analyzed in order to determine their impact on FA profile after transplantation, but no significant differences were observed.

## 4. Discussion

In the general population, it is dyslipidemia that is the major contributing factor to atherosclerosis, and CVD [17,18]. Our previous studies, among others, have demonstrated that profound lipid abnormalities in renal transplant recipients are present, and not limited solely to increased cholesterol and triglycerides concentrations, but consisting also of severe disturbances of the FA profile [6,7,11]. The exact pattern of lipidomic disorders following renal transplantation, and its associations with atherosclerosis and cardio-vascular risk, remains unknown. In this research we investigated the changes/possibility to return to a healthy profile of FA, in order to expanding our understanding of the mechanisms and the role of lipidomic abnormalities in patients after kidney transplantation. The present study confirmed and extended our earlier observations on profound deteriorations of the FA profile in patients treated with renal transplantation [6,11]. End-stage renal disease (ESRD) is a common and deleterious condition with mortality rates comparable to cancer patients [1,2,3]. Renal transplantation is by far the best treatment option for ESRD. Not only it makes the quality of life better, as compared to dialysis, but, most importantly, it impacts on patient prognosis, significantly prolonging life expectancy. However, despite improved water removal, clearance of uremic toxins, normalization of the water-electrolyte balance and endocrine disorders, profound metabolic deteriorations are still present in kidney graft recipients, and cardiovascular mortality remains the major cause of death in this patient population [19]. Lipid disorders are among these metabolic disturbances that do not normalize in ESRD subjects following renal transplantation. Atherogenic hyperlipidemia is thought to derive from ESRD itself, concomitant diseases, and the immunosuppressive treatment administered [4]. However, atherogenic lipid disorders are not limited to hypertriglyceridemia and elevated cholesterol concentration. Disorders of the FA profile are thought to contribute to CVD risk in the general population. Increased content of SFA, together with low levels of PUFA constitute acknowledged risk factors for CVD and its complications.

Taking into account the above considerations, we have undertaken the present study to widen our understanding of the FA abnormalities in transplanted patients. We took into account the time since transplantation, grouping the patients into three cohorts. Metabolic alterations of patients in the first cohort (up to one month after transplantation) are influenced by ESRD and/or dialysis therapy, as well as the recent surgery and high immunosuppression, typical for the early term post-transplant. Patients from the third cohort (longer than one year post-transplant), compose a target group of subjects with stable graft function, on low immunosuppression, with no additional confounding factors, listed for the first cohort. The second cohort (from the second month up to first year post-transplant) was an intermediate group.

Although there were some significant differences in particular SFA among the groups, the general SFA content did not differ between transplanted patients and controls. The only consistent exception were the branched chain fatty acids (BCFA), as their levels turned out to be significantly lower in kidney graft recipients. Branched chain fatty acids constitute a group derived mainly from ruminants (milk, dairy products, beef) [20]. Recent research, including our studies, suggests that BCFA promote weight maintenance and insulin sensitivity [21,22]. Both are often disturbed in transplanted patients, due to the immunosuppressive medications administered (e.g., steroids, tacrolimus). In vitro experiments, as well as animal studies point to the anti-inflammatory properties of BCFA [23,24,25]. Moreover, their anticarcinogenic effects are widely acknowledged, a feature of fundamental importance in transplant recipients as patients with a significantly increased risk of cancer development.

Although endogenous production has also been confirmed, as BCFA were found to be synthesized from respective branched chain amino acids (BCAA), e.g., valine, leucine, isoleucine, the dietary intake of BCFA is presumably the major source of these FA in the body [20]. In the present study, we did not assess the diet of the patients, which is a limitation, underlined below. However, it is probably correct to assume that the deteriorations in BCFA profile, presented above, are derived from dietary restrictions that the patients are subdued to. Patients with ESRD are advised to restrict from dairy food in order to reduce hyperphosphatemia, a constant and deleterious complication of CKD. These restrictions are mitigated following successful kidney transplantation. Hence, the lowest content of BCFA was observed in the group in their first month after surgery. During the following months and years, in association with diet normalization, BCFA tended to increase, though never reaching the levels observed in healthy controls. Moreover BCFA levels in patients seems to be associated with CVD risk, since patients with CVD had lower levels of iso-BCFA than patients without CVD.

Dietary omega-3 (n-3) FA intake and its serum content is associated with a reduced CVD risk [26]. Numerous studies, although mainly experimental in design, have demonstrated the role of n-3 PUFA in lowering plasma triglycerides, blood pressure, and indices of inflammation, as well as in improving vascular function [26]. However, the data supporting the use of PUFA following renal transplantation is scarce. Therefore, the Kidney Disease Outcomes Quality Initiative (KDOQI) guidelines do not support routine PUFA prescription in patients post-transplantation for lowering mortality or cardiovascular events [27]. The present study revealed significant decreases in most of the n-3 PUFA evaluated. However, in the inter-group analysis, a gradual normalization of n-3 PUFA content was observed. In long-term graft recipients, it did not differ significantly from the controls. As in case of BCFA, low levels of PUFA directly post-transplant resulted probably from dietary restrictions during dialysis, uremic anorexia and/or the uremic toxicity itself. Of interest, n-3 PUFA level turned out to be significantly lower in patients with impaired graft function, defined as eGFR below 45 mL/min/1.73m^2^. This stays in accordance with our previous observations of low n-3 PUFA in patients with impaired function of the native kidneys (CKD stage 4–5, dialysis) [5]. It seems that it is indeed the deterioration of kidney function that contributes to this abnormality. Moreover, in the present study, patients with indices of inflammatory state exhibited lower levels of n-3 PUFA, in comparison to subject with no inflammation. Persistent low-grade inflammation is a characteristic feature of CKD, especially ESRD, and is acknowledged as one of the, so-called, non-traditional risk factors for CVD in this patient population [28]. In transplanted patients, inflammatory state is less common. Taking into account the potential anti-inflammatory properties of n-3 PUFA, their decreased content may predispose the patients to inflammation. Obviously, this hypothesis cannot be verified on the basis of this, cross-sectional, evaluation. Similar associations were observed in the n-6 PUFA profile. The content of these FA was significantly decreased in patients immediately post-transplant, and it increased to values similar to controls in the two consecutive cohorts of graft recipients. The issue of n-6 PUFA abnormalities is difficult to interpret, as the data on their potential impact on health is debatable, especially in CKD. Some studies in ESRD patients demonstrate associations between increased n-6/n-3 PUFA ratio with carotid atherosclerosis, while others present representatives on n-6 PUFA as independently linked to reduced mortality [29,30]. At the same time, the observed curiosity was the results of the analysis of the influence of statins, taken by transplant patients, on the FA profile. Statins can stimulate the conversion of LA to DGLA, which is a precursor of anti-inflammatory oxylipins [31]. However, we did not find significant differences in CRP level between patients taking and not taking statins.

In our previous studies, we have characterized the disturbances of MUFA content in the course of CKD [6]. We have demonstrated that MUFA increase gradually with consecutive stages of CKD, that this increase is probably due to enhanced endogenous synthesis, and that it is independently associated with the CVD risk. The content of MUFA in transplanted patients was lower than in severe stages of CKD and in dialysis subjects, however, still significantly higher than in healthy controls. This was also the case in the present study. Moreover, MUFA level was significantly higher in patients with impaired graft function. Taking into account the potential influence of MUFA on CVD risk, through their contribution to hypertriglyceridemia and/or chronic inflammation, these findings might be of clinical importance [6].

Surprisingly, we have found that cyclopropaneoctanoic acid 2-hexyl is present in reduced levels in serum of Tx patients. This is seemingly contradictory to our previous studies, in which we have found elevated level of this FA in patients with CKD. Renal transplant recipients are characterized by dysbiosis [32], that may lead to decreased production of cyclopropane FA by intestinal bacteria.

The results presented in Figure 5A suggest that kidney function have an impact on serum FA profile kidney transplant recipients. However, kidney is not a key organ in FA metabolism, so this effect is rather indirect, and possibly it is related to alteration in liver function in these patients.

The primary limitation of the study is, naturally, its cross-sectional design. In such setting, we were unable to distinguish causes from consequences in the presented associations. The patient group was divided into three cohorts, depending on the time post-transplant. However, such design cannot replace a longitudinal observation of transplanted subjects. Another major limitation was associated with the lack of dietary data of the patients and controls. Our previous studies have shown only week correlations between the diet and FA profile of CKD patients. However, since in the general population, FA content heavily depends on the diet, such an analysis would definitely strengthen the value of our observations and derived conclusions.

The current study is cross-sectional in design. In order to verify the hypothesis on the impact of renal transplantation on FA, a longitudinal analysis ought to be per-formed, in which the FA profile would be evaluated in dialysis patients waitlisted for kidney transplantation, who subsequently undergo the transplantation procedure. The aim would be to longitudinally track the changes in FA profile during dialysis and dur-ing transplantation period in the same subjects.

## 5. Conclusions

This study showed that the FA profile is considerably altered in renal transplant recipients. As described above, the disturbances of the FA profile might increase the cardiovascu-lar risk in this vulnerable patient population. Obtained results might help in designing interventions (e.g., FA supplements, dietary advise) that would normalize the profile or at least ameliorate the most profound alterations. The potential efficacy of FA supple-mentation and/or nutritional education in preventing the consequences of FA disor-ders need to be assessed in future studies.

## Figures and Tables

**Table 1:** Biochemical and anthropometric characteristics of the study subjects.

	HC	Tx(1)	Tx(12)	Tx(˃12)	HC vs. Tx (1)	HC vs. Tx (12)	HC vs. Tx (˃12)	Tx (1) vs. Tx (12)	Tx (12) vs. Tx (˃12)	Tx (1) vs. Tx (>12)
Sex (F/M)	23/25	25/36	19/21	46/56	NT	NT	NT	NT	NT	NT
Age (years)	47.0 ± 2.03	51.8 ± 1.77	48.1 ± 2.46	51.7 ± 1.24	NS	NS	NS	NS	NS	NS
7-SGA (points)										
3	ND	1	ND	ND	NT	NT	NT	NT	NT	NT
4	ND	1	ND	7	NT	NT	NT	NT	NT	NT
5	ND	22	8	26	NT	NT	NT	NT	NT	NT
6	ND	26	19	47	NT	NT	NT	NT	NT	NT
7	ND	11	13	22	NT	NT	NT	NT	NT	NT
BMI (kg/m^2^)	26.1 ± 0.592	25.5 ± 0.474	25.4 ± 0.818	26.2 ± 0.493	NS	NS	NS	NS	NS	NS
FTI (kg/m^2^)	ND	11.2 ± 0.53	12.2 ± 0.70	12.7 ± 0.49	NT	NT	NT	NS	NS	NS
LTI (kg/m^2^)	ND	13.5 ± 0.38	13.4 ± 0.37	13.0 ± 0.25	NT	NT	NT	NS	NS	NS
Haemoglobin (g/dL)	14.45 ± 0.178	11.28 ± 0.169	12.30 ± 0.404	13.24 ± 0.220	<0.05 *	<0.05 *	<0.05 *	<0.05 *	NS	<0.05 *
Creatinine (mg/dL)	0.865 ± 0.025	1.98 ± 0.178	1.43 ± 0.083	1.41 ± 0.050	<0.05 *	<0.05 *	<0.05 *	NS	NS	NS
eGFR (CKD EPI) (ml/min/1.73 m^2^)	79.7 ± 1.15	47.2 ± 3.25	52.9 ± 3.49	54.9 ± 2.03	<0.05 *	<0.05 *	<0.05 *	NS	NS	NS
BUN (mg%)	15.4 ± 0.519	38.8 ± 2.732	29.8 ± 3.09	25.7 ± 1.08	<0.05 *	<0.05 *	<0.05 *	NS	NS	NS
CRP (mg/L)	2.49 ± 0.593	5.25 ± 0.955	4.19 ± 0.795	3.7 ± 0.507	NS	NS	NS	NS	NS	NS
Glucose (mg/dL)	96.7 ± 2.99	105.6 ± 3.92	93.6 ± 4.30	101.6 ± 3.75	NS	NS	NS	NS	NS	NS
Albumin (g/L)	40.2 ± 0.393	33.0 ± 0.585	35.9 ± 1.234	36.8 ± 0.643	<0.05 *	<0.05 *	<0.05 *	<0.05 *	NS	<0.05 *
Triacylglycerols (mg/dL)	119 ± 8.48	202 ± 7.75	166 ± 12.12	176 ± 14.40	<0.05 *	<0.05 *	<0.05 *	NS	NS	<0.05 *
Total cholesterol (mg/dL)	202 ± 6.22	216 ± 5.69	213 ± 9.22	198 ± 5.45	NS	NS	NS	NS	NS	NS
HDL (mg/dL)	54.6 ± 1.89	45.6 ± 1.37	49.0 ± 2.83	48.9 ± 1.59	<0.05 *	NS	NS	NS	NS	NS
LDL (mg/dL)	123 ± 5.68	138 ± 5.01	120 ± 6.88	120 ± 4.18	NS	NS	NS	NS	NS	NS
Sodium (mg/dl)	140 ± 0.325	137 ± 2.088	136 ± 3.430	138 ± 1.386	NS	NS	NS	NS	NS	NS
Potassium (mg/dl)	4.29 ± 0.044	4.42 ± 0.063	4.27 ± 0.127	4.29 ± 0.061	NS	NS	NS	NS	NS	NS

HC-healthy controls; Tx-kidney transplant patients; Tx(1)-patients up to one month after the procedure; Tx (12)-patients between 1 month and the first year post-transplant; Tx (>12)-patients in their consecutive years post-transplant. Data are presented as mean ± SEM. *p* from One Way Analysis of Variance followed by All Pairwise Comparison Tukey Test, * *p* from non-parametric Kruskall-Wallis One Way Analysis of Variance followed by All Pairwise Comparison Dunn’s Method on ranks. BMI Body Mass Index; BUN-Blood Urea Nitrogen; CKD-EPI Chronic Kidney Disease; CRP C-reactive protein; eGFR estimated Glomerular Filtration Rate; FTI fat tissue index; HDL High density lipoprotein; LDL Low density lipoprotein; LTI lean tissue index, SGA- Subjective Global Assessment; NT-not tested; ND-not determined, NS-not significant.

**Table 2:** Profile of fatty acids (%) in healthy control (HC) and kidney transplant patients (Tx) sera. Values are mean ± SEM.

	HC	Tx	*p*
8:0	0.003 ± 0.001	0.003 ± 0.001	0.685
10:0	0.020 ± 0.001	0.011 ± 0.001	˂0.001
12:0	0.25 ± 0.016	0.14 ± 0.011	˂0.001
14:0	1.17 ± 0.043	1.19 ± 0.033	0.543
16:0	23.3 ± 0.238	24.4 ± 0.129	˂0.001
18:0	7.20 ± 0.109	6.37 ± 0.068	˂0.001
20:0	0.076 ± 0.003	0.062 ± 0.002	˂0.001
22:0	0.148 ± 0.008	0.129 ± 0.005	0.002
24:0	0.140 ± 0.006	0.092 ± 0.004	˂0.001
**Total ECFA**	**32.3 ± 0.262**	**32.4 ± 0.145**	**0.959**
9:0	0.003 ± 0.001	0.004 ± 0.001	0.092
11:0	0.014 ± 0.001	0.004 ± 0.001	˂0.001
13:0	0.028 ± 0.002	0.011 ± 0.001	˂0.001
15:0	0.235 ± 0.007	0.269 ± 0.006	0.005
17:0	0.250 ± 0.006	0.262 ± 0.005	0.506
19:0	0.033 ± 0.002	0.017 ± 0.001	˂0.001
21:0	0.015 ± 0.001	0.013 ± 0.001	0.4863
23:0	0.058 ± 0.003	0.033 ± 0.001	˂0.001
**Total OCFA**	**0.632 ± 0.014**	**0.607 ± 0.010**	**0.086**
3,8,12-triM-13:0	0.012 ± 0.001	0.013 ± 0.001	0.098
iso 12-M-13:0	0.009 ± 0.001	0.010 ± 0.001	0.273
iso 13-M-14:0	0.032 ± 0.002	0.023 ± 0.001	˂0.001
iso 14-M-15:0	0.076 ± 0.003	0.040 ± 0.002	˂0.001
iso 15-M-16:0	0.086 ± 0.005	0.057 ± 0.002	˂0.001
iso 17-M-18:0	0.030 ± 0.001	0.028 ± 0.001	0.245
iso 20-M-21:0	0.004 ± 0.001	0.005 ± 0.001	0.165
**Total iso BCFA**	**0.237 ± 0.009**	**0.161 ± 0.005**	**˂0.001**
anteiso 12-M-14:0	0.046 ± 0.002	0.039 ± 0.001	0.001
anteiso 14-M-16:0	0.115 ± 0.006	0.059 ± 0.002	˂0.001
anteiso 20-M-22:0	0.009 ± 0.001	0.006 ± 0.001	˂0.001
**Total anteiso BCFA**	**0.170 ± 0.007**	**0.103 ± 0.003**	**˂0.001**
**Total BCFA**	**0.42 ± 0.014**	**0.28 ± 0.008**	**˂0.001**
**Total SFA**	**33.3 ± 0.259**	**33.3 ± 0.153**	**0.449**
14:1	0.069 ± 0.005	0.059 ± 0.003	0.001
16:1	2.94 ± 0.132	3.39 ± 0.061	˂0.001
18:1	26.4 ± 0.427	29.0 ± 0.231	˂0.001
19:1	0.026 ± 0.002	0.017 ± 0.001	˂0.001
20:1	0.169 ± 0.005	0.133 ± 0.004	˂0.001
22:1	0.042 ± 0.007	0.021 ± 0.004	˂0.001
24:1	0.223 ± 0.013	0.203 ± 0.008	0.096
**Total MUFA**	**29.9 ± 0.488**	**32.8 ± 0.250**	**˂0.001**
CPOA2H	0.161 ± 0.005	0.114 ± 0.003	˂0.001
ALA (18:3n3)	0.332 ± 0.016	0.198 ± 0.010	˂0.001
EPA (20:5n3)	1.029 ± 0.097	0.724 ± 0.032	˂0.001
ETA (20:4n3)	0.098 ± 0.004	0.054 ± 0.001	˂0.001
DHA (22:6n3)	1.12 ± 0.064	1.20 ± 0.030	0.080
DPAn3 (22:5n3)	0.290 ± 0.008	0.338 ± 0.005	˂0.001
**Total PUFA n3**	**2.87 ± 0.158**	**2.51 ± 0.062**	**0.023**
LA(18:2n6)	26.7 ± 0.560	25.2 ± 0.264	0.011
ARA 20:4n6)	5.56 ± 0.157	5.03 ± 0.088	0.004
DGLA (20:3n6)	1.152 ± 0.033	0.872 ± 0.021	˂0.001
20:2n6	0.161 ± 0.005	0.103 ± 0.003	˂0.001
DPAn6 (22:5n6)	0.058 ± 0.004	0.025 ± 0.001	˂0.001
AdA (22:4n6)	0.101 ± 0.004	0.100 ± 0.003	0.475
**Total PUFA n6**	**33.7 ± 0.578**	**31.3 ± 0.287**	**˂0.001**

*p* from Mann-Whitney Rank Sum Test. AdA-adrenic acid; ALA-α-linolenic acid; ARA-arachidonic acid; BCFA-branched chain fatty acids; CPOA2H-cyclopropaneoctanoic acid 2-hexyl; DGLA-dihomo-γ-linolenic acid; DHA-docosahexaenoic acid; DPA n3-docosapentaenoic acid n3; DPA n6-docosapentaenoic acid n6; ECFA-even chain fatty acids; EPA-eicosapentaenoic acid; ETA-eicosatetraenoic acid; LA-linoleic acid; MUFA-monounsaturated fatty acids, OCFA-odd chain fatty acids; PUFA-polyunsaturated fatty acids; SFA-saturated fatty acids. Boldface-major groups of fatty acid.

**Table 3:** Profile of fatty acids (%) in healthy control (HC) and kidney transplant patients (Tx) sera depending on the time after surgery.

Fatty Acids	HC	Tx (1)	Tx (12)	Tx (˃12)	HC vs. Tx (1)	HC vs. Tx (12)	HC vs. Tx (>12)	Tx (1) vs. Tx (12)	Tx (12) vs. Tx (˃12)	Tx (1) vs. Tx(˃12)
8:0	0.003 ± 0.000	0.003 ± 0.000	0.003 ± 0.000	0.003 ± 0.000	NS	NS	NS	NS	NS	NS
10:0	0.02 ± 0.001	0.01 ± 0.001	0.01 ± 0.001	0.01 ± 0.001	<0.05 *	<0.05 *	<0.05 *	NS	NS	NS
12:0	0.25 ± 0.02	0.12 ± 0.01	0.15 ± 0.02	0.14 ± 0.02	<0.05 *	<0.05 *	<0.05 *	NS	NS	NS
14:0	1.17 ± 0.04	1.16 ± 0.07	1.24 ± 0.08	1.20 ± 0.04	NS	NS	NS	NS	NS	NS
16:0	23.3 ± 0.24	25.5 ± 0.21	24.1 ± 0.29	23.8 ± 0.16	<0.001	0.147	0.222	<0.001	0.900	<0.001
18:0	7.20 ± 0.11	5.64 ± 0.12	6.70 ± 0.16	6.68 ± 0.07	<0.001	0.033	0.002	<0.001	1.000	<0.001
20:0	0.08 ± 0.003	0.05 ± 0.002	0.07 ± 0.003	0.07 ± 0.002	<0.05 *	NS	<0.05 *	<0.05 *	NS	<0.05 *
22:0	0.15 ± 0.01	0.10 ± 0.004	0.15 ± 0.02	0.14 ± 0.01	<0.05 *	NS	NS	<0.05 *	NS	<0.05 *
24:0	0.14 ± 0.01	0.08 ± 0.003	0.11 ± 0.02	0.09 ± 0.004	<0.05 *	<0.05 *	<0.05 *	NS	NS	NS
**Total ECFA**	**32.3 ± 0.26**	**32.7 ± 0.25**	**32.5 ± 0.33**	**32.2 ± 0.21**	**NS**	**NS**	**NS**	**NS**	**NS**	**NS**
9:0	0.003 ± 0.000	0.004 ± 0.000	0.004 ± 0.000	0.003 ± 0.000	NS	NS	NS	NS	NS	NS
11:0	0.01 ± 0.001	0.004 ± 0.000	0.004 ± 0.000	0.004 ± 0.000	<0.05 *	<0.05 *	<0.05 *	NS	NS	NS
13:0	0.03 ± 0.002	0.01 ± 0.000	0.01 ± 0.001	0.01 ± 0.001	<0.05 *	<0.05 *	<0.05 *	NS	NS	NS
15:0	0.23 ± 0.01	0.26 ± 0.01	0.27 ± 0.01	0.27 ± 0.01	NS	NS	NS	NS	NS	NS
17:0	0.25 ± 0.01	0.26 ± 0.01	0.28 ± 0.01	0.26 ± 0.01	NS	NS	NS	NS	NS	NS
19:0	0.03 ± 0.002	0.01 ± 0.001	0.02 ± 0.001	0.02 ± 0.001	<0.05 *	<0.05 *	<0.05 *	NS	NS	NS
21:0	0.01 ± 0.001	0.01 ± 0.001	0.01 ± 0.001	0.02 ± 0.001	NS	NS	NS	NS	NS	<0.05 *
23:0	0.06 ± 0.003	0.02 ± 0.001	0.04 ± 0.01	0.04 ± 0.001	<0.05 *	<0.05 *	<0.05 *	NS	NS	<0.05 *
**Total OCFA**	**0.63 ± 0.01**	**0.59 ± 0.02**	**0.63 ± 0.03**	**0.61 ± 0.01**	**NS**	**NS**	**NS**	**NS**	**NS**	**NS**
3.8.12-triM-13:0	0.01 ± 0.001	0.01 ± 0.001	0.01 ± 0.001	0.02 ± 0.001	NS	NS	NS	NS	NS	<0.05 *
iso 12-M-13:0	0.01 ± 0.001	0.01 ± 0.001	0.01 ± 0.001	0.01 ± 0.001	NS	NS	NS	NS	NS	NS
iso 13-M-14:0	0.03 ± 0.002	0.02 ± 0.002	0.02 ± 0.002	0.02 ± 0.001	<0.05 *	<0.05 *	<0.05 *	NS	NS	NS
iso 14-M-15:0	0.08 ± 0.003	0.04 ± 0.003	0.05 ± 0.01	0.04 ± 0.002	<0.05 *	<0.05 *	<0.05 *	NS	NS	NS
iso 15-M-16:0	0.09 ± 0.005	0.05 ± 0.003	0.06 ± 0.01	0.06 ± 0.003	<0.05 *	<0.05 *	<0.05 *	NS	NS	NS
iso 17-M-18:0	0.03 ± 0.001	0.02 ± 0.001	0.03 ± 0.001	0.03 ± 0.001	NS	NS	NS	NS	NS	NS
iso 20-M-21:0	0.004 ± 0.000	0.004 ± 0.000	0.004 ± 0.000	0.01 ± 0.001	NS	NS	NS	NS	NS	<0.05 *
**Total iso BCFA**	**0.24 ± 0.01**	**0.14 ± 0.01**	**0.17 ± 0.01**	**0.17 ± 0.01**	**NS**	**NS**	**NS**	**NS**	**NS**	**NS**
anteiso 12-M-14:0	0.05 ± 0.002	0.03 ± 0.003	0.04 ± 0.003	0.04 ± 0.002	<0.05 *	NS	NS	NS	NS	NS
anteiso 14-M-16:0	0.12 ± 0.01	0.06 ± 0.005	0.06 ± 0.005	0.06 ± 0.003	<0.05 *	<0.05 *	<0.05 *	NS	NS	NS
anteiso 20-M-22:0	0.01 ± 0.001	0.005 ± 0.000	0.005 ± 0.001	0.01 ± 0.001	<0.05 *	<0.05 *	NS	NS	<0.05 *	<0.05 *
**Total anteiso BCFA**	**0.17 ± 0.01**	**0.10 ± 0.01**	**0.10 ± 0.01**	**0.11 ± 0.005**	**NS**	**NS**	**NS**	**NS**	**NS**	**NS**
**Total BCFA**	**0.42 ± 0.01**	**0.25 ± 0.01**	**0.29 ± 0.02**	**0.29 ± 0.01**	**NS**	**NS**	**NS**	**NS**	**NS**	**NS**
**Total SFA**	**33.3 ± 0.26**	**33.5 ± 0.27**	**33.4 ± 0.35**	**33.1 ± 0.22**	**NS**	**NS**	**NS**	**NS**	**NS**	**NS**
14:1	0.07 ± 0.005	0.06 ± 0.01	0.06 ± 0.01	0.06 ± 0.004	<0.05 *	NS	<0.05 *	NS	NS	NS
16:1	2.94 ± 0.13	3.55 ± 0.08	3.18 ± 0.15	3.37 ± 0.09	<0.05 *	NS	<0.05 *	<0.05 *	NS	NS
18:1	26.4 ± 0.43	30.62 ± 0.39	28.3 ± 0.63	28.2 ± 0.28	<0.001	0.034	0.004	0.002	1.000	<0.001
19:1	0.03 ± 0.002	0.02 ± 0.001	0.02 ± 0.001	0.02 ± 0.001	<0.05 *	<0.05 *	<0.05 *	NS	NS	NS
20:1	0.17 ± 0.01	0.13 ± 0.01	0.13 ± 0.01	0.13 ± 0.01	<0.05 *	<0.05 *	<0.05 *	NS	NS	NS
22:1	0.04 ± 0.01	0.01 ± 0.001	0.02 ± 0.002	0.03 ± 0.01	<0.05 *	<0.05 *	<0.05 *	NS	NS	<0.05 *
24:1	0.22 ± 0.01	0.18 ± 0.01	0.23 ± 0.04	0.20 ± 0.01	NS	NS	NS	NS	NS	NS
**Total MUFA**	**29.9 ± 0.49**	**34.6 ± 0.40**	**31.9 ± 0.71**	**32.1 ± 0.31**	**NS**	**NS**	**NS**	**NS**	**NS**	**NS**
ALA (18:3n3)	0.33 ± 0.02	0.18 ± 0.02	0.20 ± 0.02	0.21 ± 0.01	<0.05 *	<0.05 *	<0.05 *	NS	NS	<0.05 *
EPA (20:5n3)	1.03 ± 0.10	0.48 ± 0.03	0.67 ± 0.08	0.89 ± 0.05	<0.05 *	<0.05 *	NS	<0.05 *	<0.05 *	<0.05 *
ETA (20:4n3)	0.10 ± 0.004	0.05 ± 0.002	0.06 ± 0.004	0.06 ± 0.002	<0.05 *	<0.05 *	<0.05 *	NS	NS	<0.05 *
DHA (22:6n3)	1.12 ± 0.06	1.09 ± 0.04	1.18 ± 0.09	1.27 ± 0.04	NS	NS	NS	NS	NS	NS
DPAn3 (22:5n3)	0.29 ± 0.01	0.34 ± 0.01	0.32 ± 0.02	0.34 ± 0.01	<0.05 *	NS	<0.05 *	NS	NS	NS
**Total PUFA n3**	**2.87 ± 0.16**	**2.14 ± 0.08**	**2.43 ± 0.17**	**2.77 ± 0.09**	**NS**	**NS**	**NS**	**NS**	**NS**	**NS**
LA(18:2n6)	26.7 ± 0.56	23.7 ± 0.39	26.1 ± 0.63	25.7 ± 0.38	<0.001	0.877	0.418	0.010	0.954	0.003
ARA 20:4n6)	5.56 ± 0.16	4.98 ± 0.15	4.89 ± 0.30	5.11 ± 0.10	NS	NS	NS	NS	NS	NS
DGLA (20:3n6)	1.15 ± 0.03	0.75 ± 0.04	0.90 ± 0.06	0.93 ± 0.03	<0.05 *	<0.05 *	<0.05 *	NS	NS	<0.05 *
20:2n6	0.16 ± 0.005	0.10 ± 0.01	0.12 ± 0.01	0.10 ± 0.003	<0.05 *	<0.05 *	<0.05 *	NS	NS	NS
DPAn6 (22:5n6)	0.06 ± 0.004	0.02 ± 0.001	0.02 ± 0.002	0.03 ± 0.001	<0.05 *	<0.05 *	<0.05 *	NS	NS	NS
AdA (22:4n6)	0.10 ± 0.004	0.12 ± 0.005	0.09 ± 0.01	0.09 ± 0.003	NS	NS	NS	<0.05 *	NS	<0.05 *
**Total PUFA n-6**	**33.7 ± 0.58**	**29.7 ± 0.44**	**32.1 ± 0.76**	**32.0 ± 0.39**	**NS**	**NS**	**NS**	**NS**	**NS**	**NS**
CPOA2H	0.16 ± 0.01	0.12 ± 0.01	0.12 ± 0.01	0.11 ± 0.005	<0.05 *	<0.05 *	<0.05 *	NS	NS	NS

Tx-kidney transplant patients; Tx(1)-patients up to one month after the procedure; Tx (12)-patients between 1 month and the first year post-transplant; Tx (>12)-patients in their consecutive years post-transplant. Data are presented as mean ± SEM. *p* from One Way Analysis of Variance followed by All Pairwise Comparison Tukey Test, * *p* from non-parametric Kruskall-Wallis One Way Analysis of Variance followed by All Pairwise Comparison Dunn’s Method on ranks. AdA-adrenic acid; ALA-α-linolenic acid; ARA-arachidonic acid; BCFA-branched chain fatty acids; CPOA2H-cyclopropaneoctanoic acid 2-hexyl; DGLA-dihomo-γ-linolenic acid; DHA-docosahexaenoic acid; DPA n3-docosapentaenoic acid n3; DPA n6-docosapentaenoic acid n6; ECFA-even chain fatty acids; EPA-eicosapentaenoic acid; ETA-eicosatetraenoic acid; LA-linoleic acid; MUFA-monounsaturated fatty acids, OCFA-odd chain fatty acids; PUFA-polyunsaturated fatty acids; SFA-saturated fatty acids. Boldface-major groups of fatty acid.

## Data Availability

Data is contained within the article or Supplementary Material.

## Supplementary Materials

The following are available online at https://www.mdpi.com/2072-6643/13/3/805/s1, Figure S1: The results of PCA based on the serum fatty acid profiles: score plot of cases (A) and variables (B) for healthy control (HC) and Tx patients more than 3 months after kidney transplantation. Table S1: Profile of fatty acids (%) in serum of healthy control (HC) and kidney transplant patients that were more than 3 months after transplantation (Tx). Tx(≥3)-patients more than 3 month after the procedure.
