# Supplementary material for: Analysis of Serum Fatty Acids Profile in Kidney Transplant Recipients
Source: Nutrients. 2021 Feb 28;13(3):805. doi: 10.3390/nu13030805 (PMC8001777; doi:10.3390/nu13030805)
Supplement: Supplementary file 1 [file nutrients-13-00805-s001.zip › supplementary material/Figure S1.docx]

**Abnormalities of serum fatty acids profile in kidney transplant recipients**

**Adriana Mika^1^, Lukasz P Halinski^2^, Tomasz Sledzinski^1,*^, Sylwia Malgorzewicz^3,4^, Paulina Wołoszyk^5^, Jolanta Dardzińska^4^, Michal Chmielewski^3^**

1. Department of Pharmaceutical Biochemistry, Medical University of Gdansk, Debinki 1, 80-211 Gdansk, Poland; adriana.mika@gumed.edu.pl (A.M.), tsledz@gumed.edu.pl (T.S)
2. Department of Environmental Analysis, Faculty of Chemistry, University of Gdansk, Wita Stwosza 63, 80-308 Gdansk, Poland; lukasz.halinski@ug.edu.pl (L.P.H)
3. Department of Nephrology, Transplantology and Internal Medicine, Medical University of Gdansk, Debinki 7, 80-211 Gdansk, Poland; sylwia.malgorzewicz@gumed.edu.pl (S.M.)
4. Department of Clinical Nutrition, Medical University of Gdansk, Debinki 7, 80-211 Gdansk, Poland; jolanta.dardzinska@gumed.edu.pl (J.D), michal.chmielewski@gumed.edu.pl (M.C.)
5. Department of Pediatric and f Internal Nursing, Medical University of Gdansk, Debinki 7, 80-211 Gdansk, Poland; paulina.woloszyk@gumed.edu.pl (P.W)

*Correspondence: tsledz@gumed.edu.pl; Tel.: +48-58-349-14-79


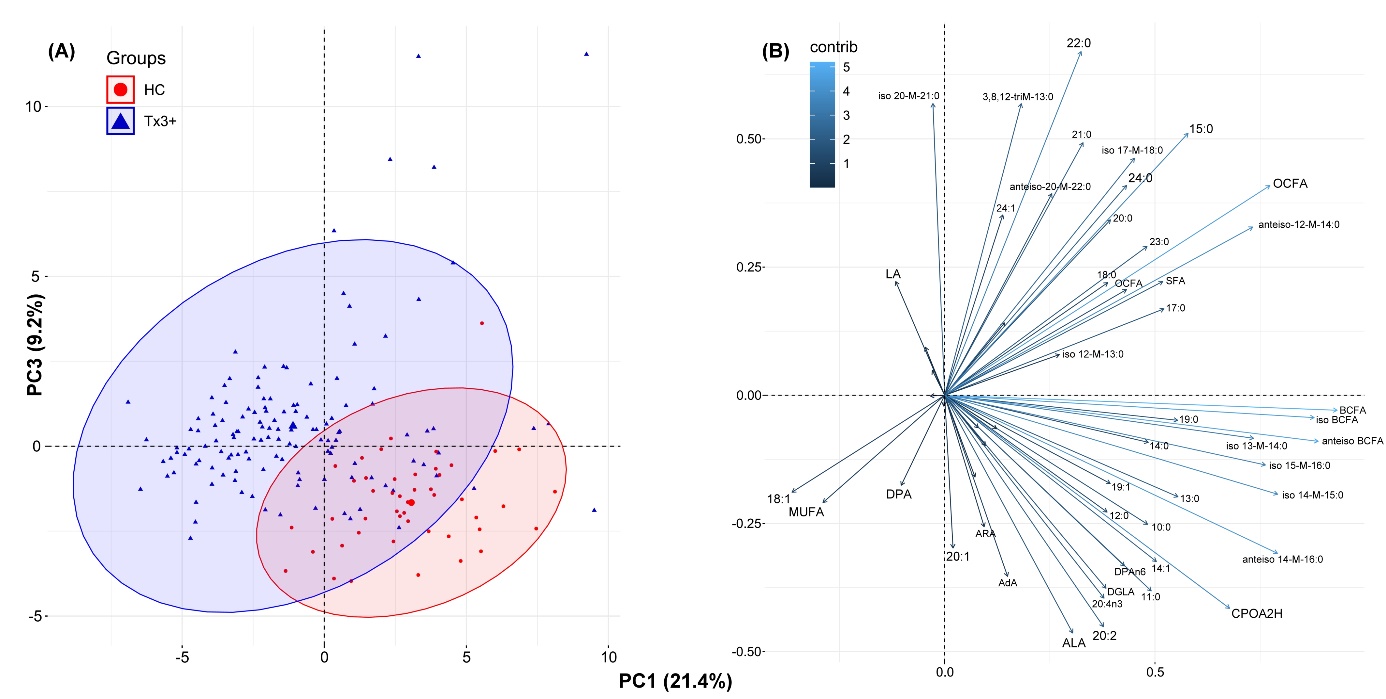


**Figure S1**. The results of PCA based on the serum fatty acid profiles: score plot of cases (A) and variables (B) for healthy control (HC) and Tx patients more than 3 months after kidney transplantation. For statistical significance of differences between groups please consult Table S1.
