# Supplementary material for: Analysis of Serum Fatty Acids Profile in Kidney Transplant Recipients
Source: Nutrients. 2021 Feb 28;13(3):805. doi: 10.3390/nu13030805 (PMC8001777; doi:10.3390/nu13030805)
Supplement: Supplementary file 1 [file nutrients-13-00805-s001.zip › supplementary material/Table S1.docx]

**Abnormalities of serum fatty acids profile in kidney transplant recipients**

**Adriana Mika^1^, Lukasz P Halinski^2^, Tomasz Sledzinski^1,*^, Sylwia Malgorzewicz^3,4^, Paulina Wołoszyk^5^, Jolanta Dardzińska^4^, Michal Chmielewski^3^**

1. Department of Pharmaceutical Biochemistry, Medical University of Gdansk, Debinki 1, 80-211 Gdansk, Poland; adriana.mika@gumed.edu.pl (A.M.), tsledz@gumed.edu.pl (T.S)
2. Department of Environmental Analysis, Faculty of Chemistry, University of Gdansk, Wita Stwosza 63, 80-308 Gdansk, Poland; lukasz.halinski@ug.edu.pl (L.P.H)
3. Department of Nephrology, Transplantology and Internal Medicine, Medical University of Gdansk, Debinki 7, 80-211 Gdansk, Poland; sylwia.malgorzewicz@gumed.edu.pl (S.M.)
4. Department of Clinical Nutrition, Medical University of Gdansk, Debinki 7, 80-211 Gdansk, Poland; jolanta.dardzinska@gumed.edu.pl (J.D), michal.chmielewski@gumed.edu.pl (M.C.)
5. Department of Pediatric and f Internal Nursing, Medical University of Gdansk, Debinki 7, 80-211 Gdansk, Poland; paulina.woloszyk@gumed.edu.pl (P.W)

*Correspondence: tsledz@gumed.edu.pl; Tel.: +48-58-349-14-79

**Table S1**. Profile of fatty acids (%) in serum of healthy control (HC) and kidney transplant patients that were more than 3 months after transplantation (Tx). Tx(**≥**3) - patients more than 3 month after the procedure.

|  | **HC** | **Tx( ≥ 3)** | **p** |
| --- | --- | --- | --- |
| 8:0 | 0.003 ± 0.001 | 0.003 ± 0.001 | 0.801 |
| 10:0 | 0.020 ± 0.001 | 0.011 ± 0.001 | ˂0.001 |
| 12:0 | 0.25 ± 0.016 | 0.14 ± 0.015 | ˂0.001 |
| 14:0 | 1.17 ± 0.043 | 1.21 ± 0.037 | 0.549 |
| 16:0 | 23.3 ± 0.238 | 23.9 ± 0.142 | 0.042 |
| 18:0 | 7.20 ± 0.109 | 6.72 ± 0.067 | ˂0.001 |
| 20:0 | 0.076 ± 0.003 | 0.066 ± 0.002 | 0.010 |
| 22:0 | 0.148 ± 0.008 | 0.142 ± 0.006 | 0.539 |
| 24:0 | 0.140 ± 0.006 | 0.099 ± 0.005 | ˂0.001 |
| **ECFA** | **32.3 ± 0.262** | **32.2 ± 0.178** | **0.884** |
| 9:0 | 0.003 ± ˂0.001 | 0.003 ± ˂0.001 | 0.002 |
| 11:0 | 0.014 ± 0.001 | 0.004 ± ˂0.001 | ˂0.001 |
| 13:0 | 0.028 ± 0.002 | 0.012 ± ˂0.001 | ˂0.001 |
| 15:0 | 0.235 ± 0.007 | 0.271 ± 0.007 | ˂0.001 |
| 17:0 | 0.250 ± 0.006 | 0.261 ± 0.006 | 0.159 |
| 19:0 | 0.033 ± 0.002 | 0.018 ± 0.001 | ˂0.001 |
| 21:0 | 0.015 ± 0.001 | 0.015 ± 0.001 | 0.938 |
| 23:0 | 0.058 ± 0.003 | 0.037 ± 0.002 | ˂0.001 |
| **OCFA** | **0.632 ± 0.014** | **0.616 ± 0.013** | **0.380** |
| 3,8,12-triM-13:0 | 0.012 ± 0.001 | 0.015 ± 0.001 | 0.016 |
| iso 12-M-13:0 | 0.009 ± 0.001 | 0.010 ± ˂0.001 | 0.090 |
| iso 13-M-14:0 | 0.032 ± 0.002 | 0.024 ± 0.001 | ˂0.001 |
| iso 14-M-15:0 | 0.076 ± 0.003 | 0.042 ± 0.002 | ˂0.001 |
| iso 15-M-16:0 | 0.086 ± 0.005 | 0.060 ± 0.003 | ˂0.001 |
| iso 17-M-18:0 | 0.030 ± 0.001 | 0.030 ± 0.001 | 0.836 |
| iso 20-M-21:0 | 0.004 ± ˂0.001 | 0.006 ± ˂0.001 | 0.002 |
| **iso BCFA** | **0.237 ± 0.009** | **0.169 ± 0.006** | **˂0.001** |
| anteiso 12-M-14:0 | 0.046 ± 0.002 | 0.042 ± 0.002 | 0.110 |
| anteiso 14-M-16:0 | 0.115 ± 0.006 | 0.059 ± 0.003 | ˂0.001 |
| anteiso 20-M-22:0 | 0.009 ± 0.001 | 0.007 ± ˂0.001 | 0.006 |
| **anteiso BCFA** | **0.170 ± 0.007** | **0.106 ± 0.004** | **˂0.001** |
| **TOTAL BCFA** | **0.42 ± 0.014** | **0.29 ± 0.010** | **˂0.001** |
| **TOTAL SFA** | **33.3 ± 0.259** | **33.2 ± 0.189** | **0.550** |
| 14:1 | 0.069 ± 0.005 | 0.057 ± 0.004 | 0.042 |
| 16:1 | 2.94 ± 0.132 | 3.32 ± 0.082 | 0.016 |
| 18:1 | 26.4 ± 0.427 | 28.2 ± 0.265 | 0.001 |
| 19:1 | 0.026 ± 0.002 | 0.017 ± 0.001 | ˂0.001 |
| 20:1 | 0.169 ± 0.005 | 0.134 ± 0.006 | ˂0.001 |
| 22:1 | 0.042 ± 0.007 | 0.024 ± 0.005 | 0.040 |
| 24:1 | 0.223 ± 0.013 | 0.213 ± 0.011 | 0.553 |
| **MUFA** | **29.9 ± 0.488** | **31.9 ± 0.296** | **0.001** |
| CPOA2H | 0.161 ± 0.005 | 0.113 ± 0.004 | ˂0.001 |
| ALA (18:3n3) | 0.332 ± 0.016 | 0.209 ± 0.010 | ˂0.001 |
| EPA (20:5n3) | 1.029 ± 0.097 | 0.842 ± 0.042 | 0.082 |
| ETA (20:4n3) | 0.098 ± 0.004 | 0.058 ± 0.002 | ˂0.001 |
| DHA (22:6n3) | 1.12 ± 0.064 | 1.24 ± 0.039 | 0.093 |
| DPAn3 (22:5n3) | 0.290 ± 0.008 | 0.334 ± 0.007 | ˂0.001 |
| **PUFA n3** | **2.87 ± 0.158** | **2.69 ± 0.081** | **0.314** |
| LA(18:2n6) | 26.7 ± 0.560 | 25.9 ± 0.328 | 0.211 |
| ARA 20:4n6) | 5.56 ± 0.157 | 5.06 ± 0.110 | 0.011 |
| DGLA (20:3n6) | 1.152 ± 0.033 | 0.933 ± 0.024 | ˂0.001 |
| 20:2n6 | 0.161 ± 0.005 | 0.104 ± 0.003 | ˂0.001 |
| DPAn6 (22:5n6) | 0.058 ± 0.004 | 0.025 ± 0.001 | ˂0.001 |
| AdA (22:4n6) | 0.101 ± 0.004 | 0.091 ± 0.003 | 0.023 |
| **PUFA n6** | **33.7 ± 0.578** | **32.1 ± 0.352** | **0.018** |

Values are mean ± SEM. AdA – adrenic acid; ALA – α-linolenic acid; ARA – arachidonic acid; BCFA – branched chain fatty acids; CPOA2H - cyclopropaneoctanoic acid 2-hexyl; DGLA –dihomo-γ-linolenic acid; DHA – docosahexaenoic acid; DPAn3 – docosapentaenoic acid; ECFA – even chain fatty acids; EPA – eicosapentaenoic acid; ETA – eicosatetraenoic acid; LA – linoleic acid; MUFA – monounsaturated fatty acids, OCFA – odd chain fatty acids; PUFA – polyunsaturated fatty acids; SFA – saturated fatty acids. Boldface - major groups of fatty acid.
